# Creating customer value from data: foundations and archetypes of analytics-based services

**DOI:** 10.1007/s12525-021-00506-y

**Published:** 2021-10-27

**Authors:** Fabian Hunke, Daniel Heinz, Gerhard Satzger

**Affiliations:** Institute of Information Systems and Marketing (IISM) and Karlsruhe Service Research Institute (KSRI), Karlsruhe Institute of Technology (KIT), Kaiserstr. 89, 76133 Karlsruhe, Germany

**Keywords:** Analytics-based services, Archetypes, Service portfolio, Cluster analysis, L8, M13, O3

## Abstract

The digital transformation offers new opportunities for organizations to expand their existing service portfolio in order to achieve competitive advantages. A popular way to create new customer value is the offer of analytics-based services (ABS)—services that apply analytical methods to data to empower customers to make better decisions and to solve complex problems. However, research still lacks to provide a profound conceptualization of this novel service type. Similarly, actionable insights on how to purposefully establish ABS in the market to enrich the service portfolio remain scarce. We perform a cluster analysis of 105 ABS and triangulate it with a revelatory case study to identify four generic ABS archetypes and to unveil their specific service objectives and characteristics. We also isolate essential factors that shape decision-making regarding the choice of adequate archetypes and subsequent transitions between them. The detailed characterization of different ABS types contributes to a more profound theorizing process on ABS as well as provides a systematization for strategic opportunities to enrich service portfolios in practice.

## Introduction

Rapid advances in information technology threaten existing product and service portfolios, drive changes in established business strategies, but also open up new opportunities for existing and new market participants (Huang & Rust, 2017; Legner et al., 2017). In these dynamic markets, the ability to expand the existing service portfolio with innovative services that exploit technological advances has become a key focus of organizations (Vargo & Lusch, 2008; Zaki, 2019).

One approach is the use of data and analytics to enable innovative services (Hunke & Engel, 2018; Lehrer et al., 2018; Troilo et al., 2017). Analytics-based services (ABS) are a novel type of service, which encompasses the application of analytical methods (‘analytics’) to data. It aims to increase customer value by supporting customers to make better decisions, solve more complex problems, and ultimately reach their goals more effectively or efficiently (Hunke et al., 2019). As an example, BASF uniquely supports farmers with ABS to increase yields (BASF Digital Farming, 2018): Using satellite images (data) of its customers’ fields and combining them with weather simulations (analytics), BASF derives current vegetation indices to predict the risk of certain plant diseases. Farmers proactively receive field-specific fertilizer recommendations including the required dosage individually calculated for each field zone. Industry experts stress that ABS hold great promise for companies to enrich their service portfolio by either achieving or sustaining a competitive advantage (Demirkan et al., 2015)—through a deeper integration in the value creation process of the customer (Saarijärvi et al., 2014) or by exploiting entirely new markets (Davenport & Harris, 2017).

Despite the research focus that the topic has received in the academic literature (Ostrom et al., 2015), it remains unclear how organizations can systematically exploit data and analytics to expand their service portfolio (Lim et al., 2018b). Recent research suggests that a systematization of opportunities provides insights into immature research topics: Möller et al. (2019) derive types of digital logistics business models to shed light on digitalization opportunities in the logistics sector, while Gimpel et al. (2018) derive types of FinTech start-ups to provide insights on how they leverage digital technologies to offer innovative financial services. Thus, systematizing with the help of dedicated service types seems a promising approach to provide organizations with actionable insights that could pave the way for new value creation opportunities in the context of ABS. Against this backdrop, the objective of this study is to contribute a systematization of ABS by revealing different ABS types and their respective purposes. Thus, the first research question states:

RQ 1: *What are archetypes of analytics-based services that can be found in the market?*

To better understand how organizations can design or extend ABS based on this systematization to specifically expand their service portfolios, the second research question further reads:

RQ 2: *Which underlying factors influence the choice of initial ABS archetypes and later transitions between them?*

We refer to archetypes as theoretical prototypes or modular service configurations that provide a systematic perspective on the different possibilities to create customer value with data and analytics. As such, they provide a common means to systematically describe and differentiate ideal configurations of ABS designs (Möller et al., 2019; Taran et al., 2015). For the analysis, we build upon cases of ABS offered by start-ups, since start-ups are often referred to as pioneers by taking advantage of new technology-enabled opportunities in their service offerings (Criscuoloa et al., 2012). We use an existing taxonomy of ABS components to classify a dataset of 105 real-world ABS offered by start-ups. Afterwards, we apply a cluster analysis according to Punj and Stewart (1983) to identify different groups of ABS cases that share similar characteristics. We complement this analysis by interviews with decision-makers accountable for managing ABS in actual start-ups to substantiate the interpretation of the resulting clusters and to derive and triangulate four different ABS archetypes. Based on these results, we map the four archetypes within a strategic framework to discuss possible pathways for organizations to establish ABS. Striving for a more comprehensive understanding of ABS and their purposeful design, we also analyze our interview series to explore essential factors that determine and influence archetype configuration and evolvement when designing ABS.

Our work contributes to the emerging discourse on ABS. First, we specify four dedicated types of ABS and describe their respective characteristics, thus laying the foundation for a more profound conceptualization of this new type of service in future research. Second, we develop a strategic framework to position the identified archetypes and hypothesize on possible strategies for establishing ABS in the market. Third, we elicit two essential factors that influence ABS design that sheds light on and may guide transition efforts towards more sophisticated ABS. Thus, also practitioners benefit from our discussion by gaining insights on how to systematically expand service portfolios with ABS. We, therefore, respond to management issues reported in the field of ABS (Hunke & Engel, 2018; Lim et al., 2018b).

The paper is structured as follows. Section 2 provides an overview of the extant literature our research builds upon. We subsequently describe our detailed research design in section 3, followed by the resulting findings in sections 4 and 5. We discuss our findings in section 6, before section 7 concludes our research, highlights its implications, reflects limitations, and proposes opportunities for future research.

## Related work

The objective of this paper is to identify ABS archetypes to provide a systematic differentiation of possible business opportunities. We first cover two fundamental building blocks for our research: We screen prior research on the conceptualization and exploitation of data and analytics in services before we then review approaches for the classification and systematization of services in general.

### Service offerings based on data and analytics

The continuously increasing amount of data, when combined with advanced analytics technologies, is considered to have great potential to expand the organizations’ business portfolio (Demirkan et al., 2015; Müller et al., 2018; Ostrom et al., 2015; Schüritz et al., 2017). Frequently termed the “datafication” or “datatization” of services, the broad field of big data and analytics has emerged as a research priority at the intersection of service and information systems research (Lehrer et al., 2018; Lycett, 2013; Ostrom et al., 2015; Schüritz et al., 2017). Large amounts of data resulting from increasingly complex and technology-driven human–human, human–machine, or machine-machine interactions in operational, customer, and other business contexts are purposefully collected, stored, and processed (Someh et al., 2019; Watson, 2009; Wirtz et al., 2018). Particularly in conjunction with cloud technologies, which offer scalable processing power and user-friendly applications, companies are increasingly able to benefit from this data (Fromm et al., 2012; Zaki, 2019). Companies expand their existing service portfolios by offering innovative services based on data and analytics, which may lead to competitive advantages (Demirkan et al., 2015; Müller et al., 2018), provide access to new markets (Davenport & Harris, 2017), and ultimately generate new revenues (Woerner & Wixom, 2015).

Against this background, several studies attempt to conceptualize the role of data and analytics in customer-facing service offerings. Chen et al. (2011) emphasize two general practices for creating new services in this context, Data-as-a-Service (DaaS) and Analytics-as-a-Service (AaaS). DaaS focuses on the delivery of raw and aggregated content. This content may stem from a multitude of possible data sources, such as publicly available social media data, or acquired private and proprietary data (Parvinen et al., 2020). The data is often distributed through an API and offers its customers a wide range of application scenarios helping them to more effectively digitally engage in their business (Delen & Demirkan, 2013; Woerner & Wixom, 2015). AaaS offers customers a wide range of customizable analysis methods that enable them to draw new insights from large amounts of data themselves. Low-barrier access to computational infrastructure allows for large-scale, complex computing ensuring successful data processing and analysis to improve or innovate business activities (Hashem et al., 2015; Lismont et al., 2019; Naous et al., 2017). These activities may range from improving decision-making processes to driving product or service innovation (Davenport & Harris, 2017; Lehrer et al., 2018), yet require a mature business understanding as well as technological and analytical readiness from AaaS customers (Dremel et al., 2020). The repertoire of analysis tools available for this purpose is usually divided into descriptive, diagnostic, predictive, and prescriptive analysis methods (Mishra et al., 2017). Both practices, DaaS and AaaS, can be offered independently or in combination in entirely new stand-alone data-driven service solutions (Hartmann et al., 2016). While this distinction is still primarily focused on business-to-business applications, Huang and Rust (2013), amongst others, add that data containing information about customers may enable a better understanding of why customers make decisions and behave in a certain way. This opens up new opportunities to deliver meaningful, customer-centric value in new IT-related (Huang & Rust, 2013) or information-intensive services (Lim et al., 2018a).

In this context, a popular topic is the creation of added value in combination with existing products using smart services. Nowadays, products are increasingly equipped with the capability for awareness and connectivity, which provides them with ‘smartness’ (Allmendinger & Lombreglia, 2005; Martin & Kühl, 2019). Smart, connected products can be amplified based on, e.g., sensor data they generate to literally ‘wrap’ meaningful product-related insights around them based on that data (Beverungen et al., 2019; Woerner & Wixom, 2015; Wuenderlich et al., 2015). Such smart services thus enrich the offering and exceed the value that is offered by the core product.

However, while a key aspect of smart services is the intensive use of contextual data, smart services require an intelligent object (Beverungen et al., 2019), which limits the concept’s general applicability in practice. Therefore, several studies examine the use of data in services in more detail to conceptualize how the organizations’ business portfolio might be affected. Schüritz & Satzger (2016) outline that the application of data in services spans a continuum of possible applications, ranging from streamlining existing service operations to building up entirely new data-driven services. Hartmann et al. (2016) develop a framework to differentiate data-driven services, which includes the kind of data source used and the (analytical) activities applied. Lim et al., (2018a, 2018b) focus on services that create value primarily through information interaction based on customer-related data and propose a chain of nine building blocks for the configuration of information-intensive services.

All these studies demonstrate that the application of data and analytics provides promising ground for the creation of new innovative service offerings. However, existing studies tend to either focus on specific application contexts, e.g., smart services amplifying smart products (Porter & Heppelmann, 2014), or examine the benefit for particular industries, e.g., manufacturing (Opresnik & Taisch, 2015)—resulting in inconsistent terminology and limited general applicability of the concepts to expand service portfolios. Similarly, studies oftentimes focus on specific data sources to conceptualize service types; e.g., IT-related services built on customer data from internal CRM systems (Huang & Rust, 2013), or smart services built on object-related data (Porter & Heppelmann, 2014). Yet, opportunities appear to be manifold and previous research emphasizes the need for service providers to actively consider different data origins and customer roles within new services when extending their service portfolios (Hunke et al., 2019). Furthermore, we found that extant research hardly discusses the (different) forms of analytical methods that might be required for value creation. The use of (sophisticated) analytics is recognized to play a key role in creating value from data (Ackoff, 1989). However, despite different conceptualizations proposed in the field, it still needs to be further investigated how data and different types of analytics—and their interplay—contribute to value in new service offerings. By zooming out of specific contexts or industries to take a holistic view on the application of data and analytics in services, we refer to *analytics-based services* as a cross-industry service type, which encompass the application of analytics to data to create customer value in new services—either as a stand-alone solution or bundled with existing products or services. Table 1 summarizes the related concepts in our context and provides the suggested definition of ABS.

**Table 1:** Overview of related service conceptualizations

Concept	Description	Contributing literature
Data-as-a-service	Service that provides its customers access to and/or aggregate a wide range of data	Chen et al. (2011), Delen and Demirkan (2013), Hartmann et al. (2016), Parvinen et al. (2020), Woerner and Wixom (2015)
Analytics-as-a-service	Service that offers its customers a wide range of customizable analytics components and infrastructure enabling them to draw insights from large amounts of data	Davenport and Harris (2017), Dremel et al. (2020), Hartmann et al. (2016), Hashem et al. (2015), Lehrer et al. (2018), Lismont et al. (2019), Mishra et al. (2017), Naous et al. (2017)
IT-related service	Service in which IT plays an essential role for customer centricity, either as a facilitator (e.g., access to customer information and customer communication) or enabler (e.g., co-creating value)	Huang and Rust (2013)
Information-intensive service	Service in which value is created primarily via information interactions rather than physical and interpersonal interactions between the customer and the provider	Lim et al. (2018a)
Smart service	Service delivered to or via an intelligent object, which is able to sense its own condition, surrounding, allows for real-time data collection, continuous communication, and interactive feedback, to provide its customers with preemptive and contextual information	Allmendinger and Lombreglia (2005), Beverungen et al. (2019), Martin and Kühl (2019), Wuenderlich et al. (2015)
Data-driven service	Service that relies on data as the key resource intending to create new value for its customers	Hartmann et al. (2016), Schüritz & Satzger (2016)
Analytics-based service	Service in which the application of analytical methods (“analytics”) to data is intended to create customer value by helping customers make better decisions, solve more complex problems, and ultimately achieve their goals more effectively or efficiently	Hunke et al. (2019)

## Systematization of analytics-based services

Systematization approaches, such as taxonomy development or archetype identification, are essential instruments for tapping into new fields of research. They contribute to structuring pre-existing research, facilitate the positioning of new contributions, and thus, support a profound theory-building process in a still underdeveloped field of research (Hambrick, 1984; Nickerson et al., 2013). IS research has adopted this approach to identify (managerially) useful generalizations and recommendations for research and practice, e.g., to better understand and manage the different types of relationships between companies and their customers afforded by services (Huang & Rust, 2013; Lovelock, 1983).

Identifying service archetypes representing generic, theoretical prototypes or modular service configurations serves research with the description of key elements of services (Hambrick, 1984; Möller et al., 2019; Taran et al., 2015). As such, they contribute to the identification of possible strategies for realizing service-business opportunities in practice. Allmendinger and Lombreglia (2005) suggest four archetypes of smart services to provide a more systematic view on business opportunities to enrich existing products. Similarly, several authors strive to systematize the manifold opportunities that digitalization, respectively the data generated in its context, offers by proposing archetypes, and thereby to more systematically manage transformation processes in practice (Weking et al., 2020; Zolnowski et al., 2016).

Current systematization approaches still focus on very specific contexts. To understand innovative approaches to services in the logistics sector, Möller et al. (2019) systematize types of novel logistics services. They thus contribute to a deeper understanding of the role of data and processing methods in the logistics sector. Comparable studies exist for the FinTech sector (e.g., Gimpel et al., 2018), or in the manufacturing industry (e.g., Weking et al., 2020). Other systematization approaches adopt a holistic perspective, but neglect to differentiate the key role of analytics for the service in greater detail, limiting their (managerial) usefulness in this regard. Hartmann et al. (2016) unveil completely new ways to conduct business solely based on data as a key resource and introduce six types of data-driven services. By differentiating the identified service types according to the underlying data source (freely available, customer provided, or tracked and generated), and to the type of performed activity (generating, aggregating, or analyzing data), they provide a basis for understanding how companies can develop completely new data-driven services that provide value. Rizk et al. (2018) develop a systematization of data-driven digital services to better understand their key elements consisting of data collection mechanisms, data utilization, insight usage, and service interaction characteristics. Applied to real-world use cases, they find that data-based services are used as either encapsulated services in larger service systems, as data visualizers, or as specialized recommenders. We argue that the emerging research field of ABS would benefit from a more general systematization of ABS unveiling different ABS types to further deepen the understanding of how data and analytics can be leveraged to systematically create new service offerings.

## Research design

In this study, we combine quantitative and qualitative research (Bryman, 2006). In the quantitative phase, we use an existing taxonomy of ABS to classify multiple real-world ABS cases from start-ups and then apply cluster analysis to group them. In the qualitative phase, we enrich our data with an additional interview series. This allows us to comprehensively interpret the identified clusters to derive ABS archetypes and to achieve a more complete understanding of ABS extending service portfolios. Table 2 provides an overview of our overall research design. In the following, we provide a more detailed description of the individual research steps and their methodological considerations.

**Table 2:** Overview of the research design

Research step	Methodological considerations	Outcome
*Quantitative phase*		
Data collection	Keyword-based collection of start-up cases from AngelList’s database (*n* = 2472) Random selection of 15 cases per keyword (*n* = 105)	Textual, case-based dataset
Data preparation	Provisional coding (Saldaña, 2009) and dichotomization of cases using an existing taxonomy as a codebook (Hunke et al., 2019) Ex-post quality check of coding using Cohen’s Kappa (Cohen, 1960)	6-dimensional, dichotomized dataset
Cluster analysis	Two-stage clustering approach: 1. Hierarchical clustering (Ward, 1963) 2. Partitioning clustering (Kaufman & Rousseeuw, 1990) and cluster validation (Rousseeuw, 1987)	Four cluster grouping of ABS cases
*Qualitative phase*		
Archetype identification and triangulation	1. Cross-table analysis of the clustering solution (Hambrick, 1984) 2. Semi-structured interviews (Bryman, 2012) and provisional coding (Saldaña, 2009) using an existing taxonomy as a codebook (Hunke et al., 2019)	Four generic archetypes of ABS
Expansion	Stepwise open and axial coding (Corbin & Strauss, 1990) to deeper understand the configuration and transitions of ABS during development	Strategy positioning map 2 key factors for configuration and transitions of ABS

### Data collection

This paper aims to derive archetypes of ABS—innovative services that provide customers with new value based on data and analytics. For this purpose, we base our analyses exclusively on ABS offered by start-ups. Start-ups tend to be the first to exploit the opportunities of new technologies for their business (Criscuoloa et al., 2012) and, unlike large organizations, start-ups often offer a single, clearly defined service. Therefore, building on ABS from start-ups as a unit of analysis seems to be adequate given the objective of our investigation.

To identify ABS cases, we draw upon AngelList's database, an online platform that enables start-ups to raise money and investors to invest in attractive business concepts. For this purpose, start-ups can advertise their projects via profiles on the platform and, thus, publish information about their company and their proposal. In addition, the companies self-categorize in the database by specifying their thematic focal points with the help of keywords.

In the first step of our collection process, we examine the available keywords and select those that can thematically be linked to ABS. We then collect all start-up cases that are related to the identified keywords, namely “analytics”, “machine learning”, “artificial intelligence”, “data mining”, “big data”, “deep learning”, “internet of things”. By collecting all cases in the first step, we aim to eliminate researchers’ selection bias (Bryman, 2012). Second, we review the identified cases to see whether they actually describe ABS. To this end, we examine whether data and analytics are described in the service description as a key aspect to create value for B2B or B2C customers. After removing duplicates, this filtering results in a final set of 2,472 cases identified as ABS.

To obtain a manageable dataset for the subsequent coding phase, we randomly select 15 use cases for each keyword. This results in a final set of 105 ABS cases with descriptions provided by the start-ups on AngelList. To expand this database with deeper and insightful information about the selected ABS, additional information about each ABS is collected from the start-up’s own website, as the level of detail of these descriptions varies enormously. This includes detailed information about the functioning of their ABS, but also information about the start-up’s evolution, insights, and beliefs that they have developed over time.

### Data preparation

**Fig. 1:**
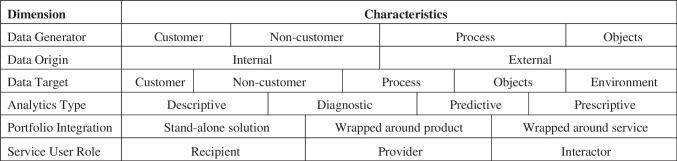
Taxonomy of ABS serving as a codebook [adopted from Hunke et al. (2019)]

Our resulting dataset consists of textual descriptions of real-world ABS. While literature provides a rich collection of possible coding mechanisms for textual case analysis, a provisional coding approach may be used in case a conceptual framework exists to serve as an underlying basis of a research inquiry (Saldaña, 2009). For this purpose, we build on research previously conducted to conceptualize the nature of ABS, which introduces a taxonomy identifying commonly shared characteristics of this service type (Hunke et al., 2019). Taxonomies are a well-established instrument to describe and analyze new phenomena using a unified classification schema (Nickerson et al., 2013). Thus, the ABS conceptualization defined in this taxonomy serves as a codebook for coding each of the 105 ABS use cases. It consists of six dimensions (cf. Fig. 1); each is represented by a distinct set of generic characteristics. A more detailed description can be found in Hunke et al. (2019).

The coding of the ABS use cases was performed by a single author. To ensure validity of the conducted coding, a random 10% sample of our dataset was individually coded by a second author in an ex-post quality check. A resulting inter-coder agreement of 88.3% as percentage agreement and 73.1% as Cohen’s Kappa (Cohen, 1960) suggests an adequate coding quality—as a Kappa value between 61 and 80% indicates a “substantial” strength of the agreement among the coders (Landis & Koch, 1977).

### Cluster analysis

Archetypes represent a typical example for a group of objects from which individual copies can emerge. Cluster analysis is a promising approach for identifying such representatives. It is a statistical technique to group similar objects according to their properties aiming to achieve high homogeneity within each cluster and high heterogeneity between objects of different clusters (Han & Kamber, 2006).

We follow the two-step procedure suggested by Punj and Stewart (1983). In the first step, agglomerative hierarchical clustering is performed using Ward’s method (Ward, 1963). Agglomerative hierarchical clustering algorithms do not require a predefined number of clusters but generate solutions for all possible cluster numbers by gradually merging the two nearest clusters in each step (Han & Kamber, 2006). To determine the distance between the individual objects, we dichotomize our dataset, i.e., each taxonomy characteristic is represented with “1” if the previous coding has identified the characteristic in the respective use case and with “0” if not. To measure the distance, we use the simple matching coefficient (Sokal & Michener, 1958), since its interpretation for binary variables well fits our context. Since cluster analysis does not provide guidance in determining the number of clusters, this preliminary analysis step helps us to obtain a first approximation of a solution examining the results in a dendrogram. It allows us to determine a candidate number of clusters and provides the opportunity to detect outliers for which cluster analysis is sensitive. In our case, four or five clusters are perceived to provide the most comprehensive insights.

In the second step, we perform an iterative partitioning clustering using the *k-medoids* algorithm. This algorithm groups objects into a predefined number (*k*) of clusters by minimizing the distance between each object and its corresponding cluster-representative object (*medoid*) for all objects in a cluster (Han & Kamber, 2006). As it uses concrete objects from the dataset as medoids, we prefer it versus the more common k-means algorithm making the results more meaningful for an archetypal interpretation. Subsequently, we validate the cluster solution using the silhouette coefficient (Rousseeuw, 1987). A solution with four distinct clusters turns out to be the stronger solution with a silhouette value of 0.41 indicating a weak, yet existing clustering structure in our dataset (Kaufman & Rousseeuw, 1990). For social science data such as ours, this is a typical result, as such data rarely exhibits strong natural groups (Hambrick, 1984).

### Archetype identification, triangulation, and expansion

To arrive at generic archetypes from our cluster solution, the grouping of objects is interpreted. We first follow Hambrick’s (1984) recommendation and inspect the characteristics’ frequency distributions of each cluster to identify the most pronounced characteristics that may serve as archetype boundaries. We perform this using a cross-table analysis.

However, archetype identification solely based on the clustering results primarily renders a static description and provides a “snapshot” of the descriptive characteristics to differentiate ABS in the current market. To gain further insights and increase data richness, we combine quantitative and qualitative research by conducting a complementary interview series to obtain both retrospective and current perceptions from those experiencing and actively shaping ABS in practice (Bryman, 2006). This interview series serves two purposes (Greene et al., 1989): First, it allows us to seek corroboration between quantitative and qualitative data, where possible, to further infuse our ABS archetype identification process (*triangulation*). Second, by expanding the breadth and scope of our data, the interviews enable us to explore factors possibly influencing start-ups’ decision-making concerning the initial choice of ABS archetypes and later transitions between them in the attempt to extend service portfolios in practice (*expansion*).

For that purpose, we conduct semi-structured interviews with senior decision-makers in start-ups that are accountable for managing ABS in practice. We purposefully sample cases of start-ups that are not part of our previous dataset, already offer an established ABS solution in the market, and that we expect to provide rich insights (Patton, 1990). During our exploratory interview study, we continuously reflect on progress in identifying and differentiating archetypes. We intentionally reach out to additional start-ups that we believe can contribute additional information to further strengthen the archetypal interpretation of our clustering results. In total, we conduct an interview series with senior decision-makers across seven different start-ups with each case (retrospectively) covering at least one ABS archetype. Table 3 provides additional information on the interviewees and the start-up cases. Following Eisenhardt (1989), such a sample size seems appropriate for our purpose, as she recommends a sample of 4–10 cases in the context of theory-building case study analyses to best balance between empirical evidence and the volume of data. Given the narrow objective of our interview series in start-ups, which typically provide a single, well-defined service and thus allow for focused analysis, this estimate is supported. We observe theoretical saturation regarding the identified archetypes as a viable means to differentiate ABS, such that incremental learning is minimal and thus closure appears to have been reached (Eisenhardt, 1989).

**Table 3:** Overview of start-up cases consulted for triangulation and extension purposes

Case (archetype)	Founded (country)	Description	Duration (in min.)	Role
Alpha (D)	2013 (Spain)	Alpha evolved into a leading provider of real-time data and analytics in the Spanish retail industry. Using a proprietary indoor geo-positioning algorithm and profound ML capabilities, Alpha is able to determine patterns of consumer behavior in grocery stores and, thus, provide useful insights and recommendations for their customers	50:02	Founder/CTO
Beta (C)	2016 (Germany)	Beta provides a customer loyalty system for more than 1000 business partners (e.g., coffees, restaurants, bakeries). They generate customer data with a bonus point system and use analytics to create user profiles and segments and, subsequently, create customized marketing campaigns. Their partners can roll out those campaigns automatically and, thereby, significantly increase their revenue	38:49	Founder/CEO
Gamma (C)	2017 (Germany, Switzerland)	Gamma offers automated revenue management for smaller hotels and serviced apartments to increase their revenue by 15–20%. To achieve this, they leverage their specific domain knowledge and analytical capabilities to offer affordable pricing recommendations and enable customers to automatically adjust their pricing strategy based on real-time data	30:33	Founder/CEO; Data Scientist
Delta (C)	2017 (Germany)	Delta develops intelligent software for demand-oriented personal planning, with a main focus on the catering and restaurant industry. With an automated demand forecast based on both internal sales data as well as externalities (e.g., weather), their solution can be used to optimize staff scheduling. Using Delta’s ABS, customers can react to unforeseen events in real-time and, thereby, increase their turnover	45:18	Head of Product
Epsilon (D)	2018 (Germany)	Epsilon provides analytical software services based on decentralized machine learning applications, with a main focus on the manufacturing industry. Epsilon’s ABS enables its customers to develop analytical models without the necessity to centralize their end customers’ sensitive usage data. Novel forms of business thus become possible without the risk of data compromise	59:54; 53:29	Founder/CEO; Founder/CTO
Zeta (A)	2017 (Germany)	Zeta offers a data marketplace for telemetric vehicle data connecting car manufacturers as data suppliers and a variety of data consumers including insurance providers and fleet managers. They provide both a standardized API for data access and the infrastructure for data transfer to end-users. Additionally, they offer support services such as factorizing and processing micro-payments for consumed data	56:20	Head of Sales
Eta (B)	2020 (Germany)	Eta provides a service for connecting innovation managers based on their overlapping interests and complementary competencies. After registering for the network, a user can prioritize profiles of other participants with whom they would like to be connected on a one-to-one basis. Using analytics-based matchmaking, the network connects participants	53:31	Founder/CEO

We analyze the interviews, again, using the established ABS taxonomy (Hunke et al., 2019) to capture the practitioners’ statements reflecting ABS characteristics. Afterwards, we search for relationships between and among the archetype boundaries and thus continuously substantiate the insights gained from the cross-table analysis by repeatedly comparing and triangulating them, where possible, with the results of the interview analysis (Jonsen & Jehn, 2009). We perceive this as helpful, for instance, to better sense and articulate the overall objective of a given archetype, complementing the preliminary results based on the descriptive characteristics.

Next, we focus on expanding the scope of ABS. Herein, we focus on factors that drive and influence the design and transitions of ABS as a means of enriching service portfolios. For that purpose, we first conduct an open coding on the interviews to inductively identify relevant first-order concepts besides descriptive characteristics that practitioners mention concerning their current ABS solution and its respective development (Corbin & Strauss, 1990). We use adequate terms used by the interviewees or descriptive phrases for this purpose. By starting with an open coding approach, we minimize both researchers’ subjectivity and possible preconceptions that may arise from the subsequent steps for interpreting the data, thus contributing to the overall confirmability of the research. Second, we apply axial coding and condense the identified concepts into second-order themes based on the previously identified archetype boundaries (Corbin & Strauss, 1990). To this end, we search for relationships between and among identified topics. Subsequently, we aggregate similar themes to sharpen factors possibly influencing the design and transitions of ABS during their development.

## Four archetypes of analytics-based services

The cluster analysis identifies four different clusters, consisting of 24–28 cases each. Each cluster exhibits different centers along the characteristics of the ABS taxonomy. The resulting cross-table provides an overview of the frequency distribution of characteristics for each archetype (cf. Table 4).

**Table 4:**
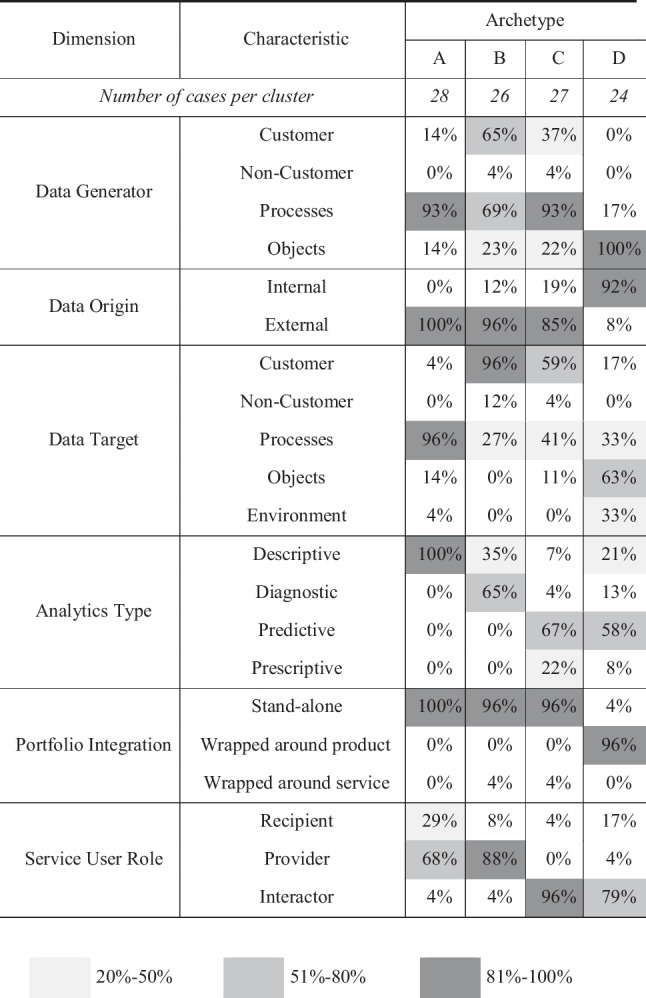
Characteristics’ frequency distributions for each archetype

Based on these findings, we conceptualize four archetypes of ABS that reveal the objectives that market-pioneers pursue when they provide services that create new value for their customers from data and analytics—(1) ABS making data usable to customers, (2) ABS delivering data-based insights, (3) ABS providing data-based recommendations, and (4) ABS enabling novel ways to conduct business. Table 5 summarizes the four archetypes we identified during our analysis, highlights their separating characteristics, and provides a typical service instantiation in practice.

**Table 5:** Summary of the identified archetypes

Archetype		Separating characteristics	Typical applications
A	Making data usable to customers	Descriptive analytics to make data useful/accessible for its customers Data created by customers’ processes or by publicly available sources Customer involvement ranging from a (reactive) recipient to an (active) data provider	Aggregated reports, dashboards, APIs
B	Delivering data-based insights	Diagnostic analytics to deliver supportive, actionable insights Customer data generated by dedicated business process or elsewhere Customers involved as data providers	Target benchmarks, meaningful alerts
C	Providing data-based recommendations	Predictive analytics to provide pre-emptive, action-inspiring, tailored decision support Customer data generated by dedicated business process or elsewhere Deep integration of and high interaction with customers	Automated situative recommendations
D	Enabling novel ways to conduct business	Predictive analytics to enable improved/novel ways to conduct business Newly created data sources to derive highly unique and customer-specific information Data-collecting objects deeply integrated into customers’ workflows requiring close interaction	Workflow integrated sensor-based IoT objects (i.e., smart products and services)

Below, we describe each archetype in detail with respect to the overall objective for the value proposition as well as the distinguishing characteristics and illustrate it with a real-world example.

### Archetype A: making data usable to customers

The first cluster characterizes ABS that aim to make existing data sources usable to their customers. While these customers oftentimes are aware of or might have access to large amounts of data in their business context, raw data itself is not adding value to them. Thus, this ABS type processes data in ways so that customers can access it and integrate it more easily into their daily activities. One example of a start-up offering this ABS type is *Rollbar*. Rollbar provides a real-time error reporting system and continuous deployment monitoring for software development teams. By integrating Rollbar into the customer’s local development architecture, it collects all incident tickets that are created for detected errors. This data—which, potentially, is available independently from Rollbar—is aggregated and visualized in a real-time dashboard. Additionally, it automatically links subsequent bug reports to the respective ticket and calculates the correlation of errors with previous occurrences. Providing aggregated information that team leaders can build on, the service allows to capture errors earlier during development and, thus, creates value by improving software delivery processes across the entire development lifecycle.

Typical for this type of ABS is the use of process data (93%), e.g., business KPIs or production data. That data is generated by the ABS customers (external data origin: 100%). Other examples for external data origin are data collected from publicly available sources. The data is processed using descriptive analytics (100%) to aggregate or visualize insightful information. These insights predominantly target customers’ business processes (96%). Thus, customers mostly engage with archetype A by providing the service-relevant data (68%) themselves. In case the data originates from publicly available data sources, the customer merely receives the aggregated information without any further engagement with the ABS (29%). Typical applications in this cluster constitute aggregated reports, dashboards, or APIs, which provide customers with the opportunity to make better decisions based on data. In the case of Zeta, for example, the ABS provider interacts with both “data providers [car manufacturers] and several potential data consumers in the mobility sector such as car insurers, fleet managers, […] or automotive associations”. They provide an API and other services such as consent management for these two types of service users allowing Zeta to “build a data pipeline” between them.

### Archetype B: delivering data-based insights

The second cluster describes ABS that aim to create new value by delivering meaningful insights to their customers based on data. In contrast to the previous archetype, which aims at enabling customers to use data on their own behalf, this archetype also “digests” the data for the customer. One example of a start-up offering this ABS type is *UBiome*. Ubiome provides a healthcare ABS that allows customers to understand their microbiome with the ultimate goal to improve their lifestyle. Ubiome provides a self-sample kit, which their customers use at home. The sample is sent to Ubiome, where it is analyzed using advanced statistical techniques. A personalized diagnosis report is prepared comparing the results with a health reference range, and individual insights are provided based on which the customer can improve his everyday life, e.g., via healthier nutrition. Archetype B uses more sophisticated, diagnostic analytics to deliver actionable insights customers can apply, e.g., to make more informed decisions.

ABS in this cluster use data that is generated by dedicated business processes (69%) or data that is generated elsewhere by the customer (69%). Archetype B relies on data that originates externally (96%). The data is predominantly analyzed to identify individual insights about customers (96%). This is achieved by using diagnostic analytics (65%), i.e., it not only highlights *whether* something happened but also delivers insights into *why* something happened. Similar to the previous archetype, the customers mainly engage in the ABS by providing the relevant data themselves (88%). Typical applications in this cluster comprise targeted benchmarks or meaningful alerts within customer processes. Within our interview sample, we identified case Eta as representing this archetype since their ABS provides analytics-based “alerts” in the event of a successful match within their customer network.

### Archetype C: providing data-based recommendations

The third cluster describes ABS that aim to provide customers with meaningful, contextual recommendations for actions to solve problems. Business value is really unlocked from data when critical insights are not only gained but followed by immediate actions applying that new knowledge. Archetype C processes data in a way that allows to predict possible outcomes and to make recommendations to the customer inspiring immediate action and supporting the customer’s decisions. One example of a start-up offering this ABS type is *Proximus*. Proximus uses advanced machine learning models to analyze consumer behavior in brick-and-mortar stores. They identify popular product dependencies among consumers and predict future revenue streams on a daily basis to recommend better store layouts or product bundles. Highly engaging with their customers via an online platform, Proximus’ recommendations inspire immediate actions for decision-makers that lead to higher sales on a single-store level.

Typical for this type of ABS is the use of process data (93%), which is occasionally enriched with customer-generated data (37%). The data mainly originates externally (85%). However, in this cluster start-ups also start using own, internally generated data (19%) such as self-built machine learning models or relevant self-collected data. This ABS archetype is primarily intended to derive customer-specific, i.e., individually tailored insights (59%). Distinctive for this cluster also is the use of advanced analytics using predictive (67%) or prescriptive methods (22%) to derive the required insights. The recommendations provided by ABS of archetype C are highly situative and tailored to the customers’ individual needs. To achieve this, this type of ABS requires deep integration and a high degree of interaction with the customer (96%). In the case of Delta, for example, their ABS accesses the sales data used from their customers’ point-of-sales systems (process data) and combines it with other customer data such as employee information to provide analytics-based recommendations on optimal staff planning.

### Archetype D: enabling novel ways to conduct business

The fourth cluster describes ABS that aim to create new value by enabling truly new ways to conduct business for their customers. In contrast to the previous archetypes, archetype D creates completely new data sources that contain customer-specific information. Thereby, this type of ABS creates new opportunities to identify meaningful insights and to make purposeful recommendations for their customers. Typical applications for this archetype are sensor-based IoT objects that are integrated into the customer’s workflow to deliver new data, insights, and ultimately provide ground for improved ways to conduct business. One example of a start-up offering this ABS type is *Skycatch*. Skycatch provides its customers, construction management firms, with a self-developed drone, which allows them to digitize large construction sites using 3D-mapping technology. Skycatch seamlessly integrates this data, which is continuously updated, e.g., on a daily basis, into the customers’ own data models using a cohesive data suit. This integration allows their customers to renew existing workflows, reaching previously unattainable gains and efficiencies, e.g., by controlling contractors’ billing for removing dirt through calculating the dirt volume based on 3D image.

Typical for this type of ABS is the usage of data generated by objects (100%), e.g., sensor data. Thus, ABS providers of this archetype draw upon their own, internal data (92%). The data is analyzed regarding the object’s own condition and state (63%) or the customer’s general business processes (33%). These insights are predominantly derived using predictive analytics (58%). Similar to archetype C, these services use customer-related data sources resulting from deep customer interaction (79%). The ABS provided by Alpha, for example, uses shopping carts equipped with sensors to enable supermarkets to “become data-driven” and use “analytics as a foundation for their decision-making” (Alpha).

## Enriching service portfolios with analytics-based services

Based on the four archetypes of ABS that we conceptualize in the previous section we elaborate on the decision-making process of ABS providers to successfully expand their service portfolio with ABS. To this end, we first map the identified archetypes in a framework to deepen the (strategic) understanding and the relationships between them. Second, we present essential factors that influence the choice of initial ABS archetypes or subsequent transitions between them in practice.

### Strategy positioning map for analytics-based services

To contribute to systemizing the field of ABS and to develop an understanding of how these four archetypes relate to each other, we develop a strategy positioning map along two key dimensions: First, Rust and Huang (2014), amongst others, describe how services can evolve over time. Service offerings expand from static “selling services” to interactive “co-creating services” as the relationship between the service provider and the customer becomes stronger over time. Building on that, we define a relationship axis, ranging from “transactional” to “relational”, to capture the service’s degree to which a continuing, stronger relationship is maintained and the ABS is more deeply embedded in the customer’s systems and working habits. Second, we noted that our results suggested a tendency towards a steady transition from external to internal data sources that were used in the different archetypes suggesting a shift from using rather commonly available data to uniquely generated data. We define a data uniqueness axis, ranging from “common” to “unique”, which captures the degree of uniqueness of the data used in the ABS.

**Fig. 2:**
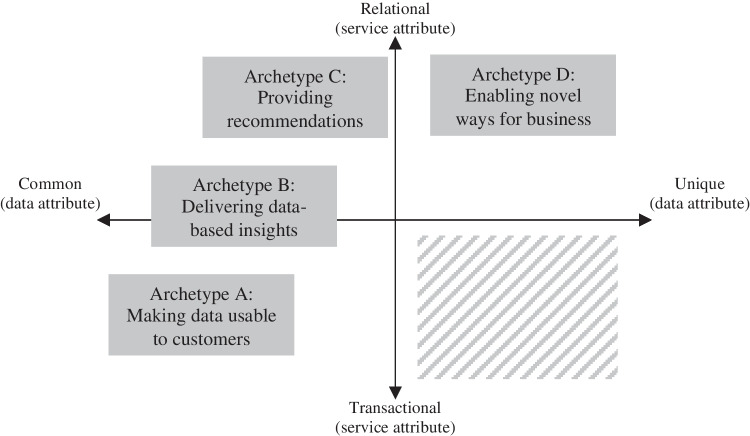
Strategy positioning map of ABS archetypes

Figure 2 shows the position of the four archetypes using these two dimensions in the strategy positioning map. Archetype A is based on common data and rather static customer interaction, as the service-relevant data is also easily applicable for potential competitors and the actual value is created downstream by the customer. In archetype B, the interaction with the customer is more pronounced since the service is more tailored to the individual customer and the service provider more strongly contributes to the joint value creation process. This results in the application of more demanding analytics in the service. Archetype C is characterized by a strong, continuous, and interactive customer relationship, as the service is deeply embedded in the customer’s processes. While customer-provided data could, potentially, also be applied by competitors, archetype C integrates its own data, analytical models, and experiences to create individual, more meaningful recommendations increasing the uniqueness of the underlying database for the service. Archetype D resembles similar relational characteristics compared to archetype C. Yet, archetype D strongly builds on new, self-generated data sources resulting in a high uniqueness of the data. Both archetypes C and D rely on sophisticated, predictive analytics to reach the intended service objective.

### Factors shaping analytics-based service configuration and evolvement

**Fig. 3:**
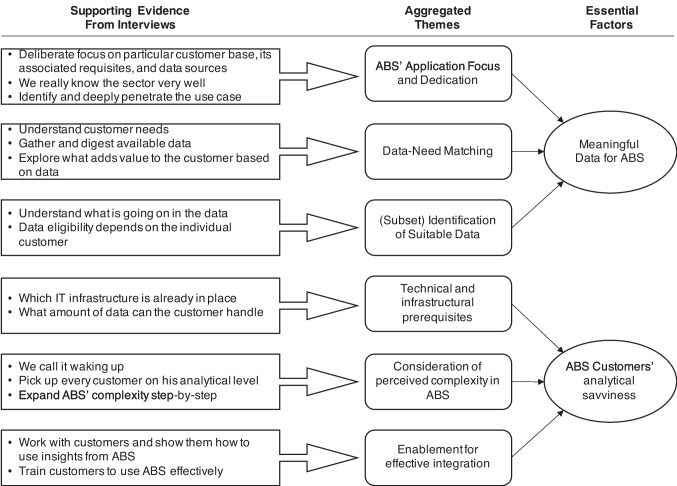
Two essential factors shaping ABS configuration and strategic evolvement

From our qualitative study, we also identify essential factors that are key for decision-making during the development of ABS and for managing ABS in service portfolios. As such, they determine and influence the configuration of ABS and transitions between ABS archetypes as customer relationship deepens and data uniqueness increases, e.g., to strategically evolve from one archetype to another in the attempt to increase customer value (cf. Fig. 2). Figure 3 illustrates our final data structure of the qualitative interview analysis, which led us to isolate two key factors. First, ABS require *meaningful data* rather than simply large amounts of (unique) data. Second, ABS need a deep customer understanding regarding their *readiness to understand and integrate ABS* in own processes to evolve ABS to more sophisticated ones. In the following, we elaborate on these two factors and provide more detailed evidence from our interview analysis.

#### Analytics-based services require meaningful data rather than big data

Our findings support the notion to reflectively and purposefully collect datasets that are unique to ultimately build differentiating and value-adding ABS. The start-ups we spoke with all deliberately focus on a specific use case to narrow down the application domain for their ABS. Although their ABS would be quite transferable to other areas, they use this focus to ‘dig deeper’ into their use case to identify and, ultimately, address specifics and requirements. CTO of case company Epsilon noted in this regard:

“So, what we do is, we actually do not broaden our focus, but we become more specialized now. That means we focus on industrial machinery [in the manufacturing industry] and we now focus heavily on the specific requirements of this customer group. This includes, for example, the special features regarding decentralized processing of data. [...] So, we become familiar, so to speak, with countless dimensions that are relevant—and I think that is also a competitive advantage, that we now have a deep understanding of this segment. Primarily, this has nothing to do with our technology, [...] but rather with the question of who actually benefits.” (Epsilon)

By diving deeper into their application domain as well as its associated specifics and requirements, startups pursue what we refer to as data-need-matching. The perceived value of data (and derived insights from them) can vary widely—even among potential customers in the same ecosystem, as Head of Sales illustrated in the case of zeta:

“The same data point [, e.g., a car’s mileage,] might not have the same value to an insurance company as it does to a leasing company or a repair shop.” (Zeta)

That makes it even more important to understand customers’ intentions and associated expectations from the ABS to determine what kind of data and analytics application might actually be meaningful to them. The start-ups emphasized deliberately taking the time to really understand their customers’ needs, and desires in the context of the identified use case. Head of Product from case company Delta described the approach to data-need-matching as follows:

“It’s really about communication. Essentially, I try to reflect on what customers want in a way that’s even a little bit detached from our solution expertise. Usually, customers come to me and say, ‘I need this button to do that’, then we go deeper into the dialogue and say, ‘Hey, what do you need this for, walk me through it’. […] It’s hard for customers to make specific requests. They can only say ‘Ok, I need this.’ I try to understand their processes as much as possible upfront and really focus on their needs and ask again. In the beginning, we have a lot of loops like that.” (Delta)

They use these insights to understand early on what data really matters to sharpen their analytics efforts. Instead of investing time, resources, and energy into collecting large amounts of data, we learned that start-ups consciously focus on identifying relevant (sub-) datasets to build their ABS around meaningful data and analytically derived insights that their customers truly value. They achieve this either by identifying specific datasets beforehand or by deliberately prioritizing specific aspects in their data when applying analytics.

“Nowadays, people like to gather information [as much as possible]. You also need the resources, the time, and the expertise to analyze that data. [..] But you need to understand what is going on in there.” (Alpha)

“The data we use [, i.e., current hotel room prices,] could generally collect a competitor. But our data is unique because we actually have a kind of intelligence, that determines from which sources we collect data, specifically. So, we do not take into account all market data but only that of pre-selected market participants—we have put a lot of effort into this.” (Gamma)

#### Analytics-based services evolve with customers’ analytical savviness

Our research suggests that adding technical features, i.e., more (sophisticated) analytics, to ABS appears not to be a promising strategy per se. Instead, we found that the level of analytical sophistication applied within ABS depends on and should evolve with *customers’ analytical savviness*. We characterized customers’ analytical savviness as their readiness to comprehend and apply analytics expertise themselves and incorporate analytical results into their workflow practices.

Important prerequisites to this end are technical and infrastructural conditions that prevail at the customer site. The ability to technically process data or integrate analytical models into own processes must be considered on a per-customer basis. Otherwise, poorly developed or missing competencies can be a pitfall by reducing the perceived added value of the ABS, and thus, need to be assessed when developing or evolving ABS—as the following statements indicate:

“It comes down to what they really have in place. Is a cloud solution already integrated? How capable is the industrial PC that is next to the machine? Do they have a deployment solution? Can they deploy a docker container or not? Some have the capability, some do not. So technologically, there is a tremendous difference [between customers].” (Epsilon)

“There are vehicle manufacturers [, providing the data for Zeta’s ABS,] that offer live streaming of data, which gives me hundreds of new data points every minute for the same vehicle. [...] That’s also a challenge for many customers, working with such a large amount of data. Some are overwhelmed. You need the IT infrastructure to do it, data analysts to understand the data, etc.” (Zeta)

Besides assessing the technical and infrastructural requirements, start-ups also described to actively consider the complexity of their ABS as perceived by their customers. We learned that ABS providers carefully assess customers’ analytical sophistication while considering to further evolve their ABS. ABS may create completely new value by improving customers’ performance, or by enabling better decision-making. Yet, they require the customer to have a certain level of expertise to unlock this potential value and, ultimately, turn it into real value (-in-use) (Grönroos & Voima, 2013). To this end, our interviewees emphasized the need to align the analytical complexity applied in ABS with the analytical capabilities required to implement them. More complex ABS, e.g., based on more comprehensive machine learning models, increased automation, or further-reaching decision-making powers, should only be deployed once the capabilities and trust on the customer side had been strengthened. Complex analytical applications at the beginning of a new ABS relationship between the provider and the customer were described as rather obstructive. Instead, they described a growing analytical savviness on the customer side as the relationship progresses along with the customer literally “waking up” allowing ABS to evolve:

“We call it ‘waking up’. [...] We really try to pick up every customer on his level [i.e. his analytical expertise]. […] That’s where we are very close to our customers.” (Delta)

“We can adjust the prices for our customers right away [e.g., on online booking platforms]. We want to make our service as simple as possible for the user and as automated as possible. But that requires a lot of machine learning in the background. In fact, we do not use any of the typical buzzwords when presenting our solution. It is important to us that it makes ‘click’ for the customer. In the beginning, they still do [price adjustments for hotel rooms based on our suggestions] manually, because they just want to verify it. But once the trust [in the ABS] is there, most of them say, ‘Alright, do it automatically [for us]’.” (Gamma)

To understand the customers’ prerequisites, practices, and how they integrate ABS into existing processes, the start-ups we talked to create dedicated touchpoints. This allows them to both build closer relationships and enable customers to effectively integrate the ABS:

“[If we] focus on—ok, I give you a dashboard, a heat map, whatever, and you have to figure it out yourself—in our experience, if you do that, [the customers] will not use that. You need to work with them and show them how to use the insights.” (Alpha)

“We talk to our new customers on the phone once a week in the beginning and later once a month. [...] Our customers really appreciate this relationship, and we benefit from the fact that we can train our customers to use our tools [i.e., the ABS solution] effectively.” (Beta)

Such close relationships enable them to pinpoint their customers’ analytical savviness and, based on this, to identify meaningful application scenarios to further evolve their ABS. Our interview series mostly took place between June and August of 2020—i.e., about three months after the pan-European outbreak of the COVID-19 pandemic and the associated onset of restrictions due to policy intervention measures (e.g., distance regulations or guest restrictions in restaurants). Surprisingly, each of the start-ups we interviewed already shared details of deliberate developments and implementations of their ABS that had taken place to best support their customers. One could argue that start-ups like the ones we spoke with are characterized by a fundamentally more agile service development and corporate culture that fosters such rapid response. However, our interviewees emphasized that being close to their customers through dedicated touchpoints and gaining deep customer knowledge allowed them to accurately assess their customers’ analytics savviness and thus the level of ABS complexity they could entrust to their customers to effectively ensure value-in-use and thus act quickly.

## Discussion

This research examines ABS as a means of creating customer value from data and analytics. It identifies four distinct archetypes that shed light on the objectives market-pioneers pursue when offering such services to create novel value for their customers, namely, to make data usable to customers, to deliver data-based insights, to provide data-based recommendations, and to enable novel ways to conduct business. Using data and analytics in service offerings as a means to create new customer value has recently become a popular strategy by organizations (Davenport & Harris, 2017; Demirkan et al., 2015) and is being actively explored by academics (Hunke & Engel, 2018; Ostrom et al., 2015). The four identified ABS archetypes partially echo findings from the existing literature and thus connect previously detached but related research strands. Archetype A, which aims to provide access to existing data to ultimately enable its customers to engage with that data in a more meaningful way, generally reflects the DaaS paradigm (Chen et al., 2011) and partly resembles “data-aggregation-as-a-service” activities as described by Hartmann et al. (2016). These studies also recognize that companies might create value by aggregating data from multiple sources of their customers and distributing it via an API or dashboard-based visualizations, afterwards. Both archetypes C and D, which rely on sophisticated and deeply integrated analytics to enable novel business activities, partly resemble the AaaS paradigm (Chen et al., 2011). Extant AaaS literature similarly identifies possible business opportunities by providing advanced analytics for data streams stemming from sensors, intelligent objects, and connected machines (Delen & Demirkan, 2013; Naous et al., 2017). Archetype D ties into research on smart services and their respective service systems (Allmendinger & Lombreglia, 2005; Beverungen et al., 2019). Aiming to enable its customers to conduct novel ways for business, these ABS often implement smart products to collect customer-specific, highly unique data within newly established service systems to provide sophisticated analytics. Integrating such ABS into their portfolio may allow companies to tap into new business opportunities, e.g., by turning into a smart platform provider (Beverungen & Kundisch, 2020), or by participating in larger service ecosystems (Papert & Pflaum, 2017). We embed these four archetypes in a single framework, the strategy positioning map, and use it to unravel distinct opportunities for how organizations can use data and analytics to develop and prospectively evolve novel services. Thus, we extend previous research on technology-driven service strategies in general (Huang & Rust, 2017) and data-driven application scenarios in particular (Hartmann et al., 2016; Schüritz & Satzger, 2016).

To determine and influence the development and management of ABS in service portfolios, striving for meaningful data as the basis of ABS emerged as a key driver. Exploring big data—most commonly characterized by its huge volume, high velocity of real-time information processing, and a wide variety of data sources (Lycett, 2013)—to discover opportunities for service innovation and develop new service offerings has emerged into a focal point of service research (Günther et al., 2017; Lehrer et al., 2018; Ostrom et al., 2015). Gradually, it is being perceived as equivalent to physical goods and capital assets (Porter & Heppelmann, 2014). However, experts note a tendency towards an unreflective, technology-driven ‘catch-all-you-can’ approach to large amounts of data, which hinders the fruitful exploitation of business opportunities (Ross et al., 2013; Yoo, 2015). In our research, decision-makers deliberately manage to drill down to identify relevant subsets to solely build on data that is meaningful to ABS customers. Data exploration and preprocessing activities are quite common practices for analytics applications. However, developing and strategically evolving ABS requires actively combining them with practices such as customer needs or experience modeling (e.g., Teixeira et al., 2012). This is crucial to successfully match relevant data and customer needs in value-adding ABS—implying the need to integrate project teams’ expertise in this regard (Joly et al., 2019).

Service is a key context for the application of technology. Formerly anchored in a ‘low-tech, high-touch’ paradigm, services are increasingly being reshaped by technology (Bitner et al., 2000; Huang & Rust, 2018)—particularly, adding analytical features to existing services has lately been considered a top priority for large organizations (Davenport, 2018). Still, customers typically demand meaningful outcomes, not overly complex services. In our research, successful ABS are closely connected to customers’ analytical savviness, which enables them (if sufficiently developed) to effectively integrate and deploy ABS outcomes. As organizations strive to strategically evolve their ABS from one archetype to another, e.g., in an attempt to increase customer value or to leapfrog competition (Davenport & Harris, 2017), they should continuously assess their customers’ readiness to comprehend, apply, and integrate analytical expertise into workflows to unlock the potential value of ABS and, ultimately, translate it into real value (-in-use) (Grönroos & Voima, 2013). Thus, as a second key driver to determine and influence the development and management of ABS, our research indicates that ABS should deliberately be evolving with customers’ analytical savviness.

## Conclusion

With the digital transformation gaining momentum, organizations increasingly explore how to expand their existing service portfolio using ABS. Yet, conceptual knowledge on this novel service type remains limited, and IS literature misses to provide foundations and actionable insights on how such a portfolio enrichment can be achieved systematically. Building on a data sample of 105 ABS use cases that are offered by start-ups in the market, using an established clustering procedure, and conducting complementary interviews with senior decision-makers accountable for managing ABS, we derive four distinct archetypes of ABS that provide initial empirically grounded evidence how ABS create new customer value. These archetypes suggest that organizations may enrich their existing service portfolio using ABS by either (1) making data usable to customers, (2) delivering data-based insights, (3) providing data-based recommendations, or (4) enabling novel ways to conduct business. Each archetype is described by a set of distinct characteristics that unveils the unique interplay of data, analytics, and customer integration for each type. Moreover, we establish a deeper understanding of decision-making during ABS development and ways to purposefully extend service portfolios with ABS. We introduce a strategy positioning map to disentangle ABS archetype configurations along common dimensions and identify two essential drivers that may guide targeted transitions from one archetype to another to systematically evolve ABS for greater impact.

### Implications for research and practice

This study offers several theoretical implications that contribute to a deeper understanding of ABS and, thus, to the ongoing debate on creating value in service offerings by means of data and analytics. Researchers certainly discuss data and analytics as a means to gain or sustain competitive advantage (Davenport & Harris, 2017; Opresnik & Taisch, 2015). Our research complements this discourse by providing yet required empirical insights that show how organizations translate data and analytics into innovative, customer-facing, and value-creating business opportunities (Günther et al., 2017). Thereby, we respond to research called for by, e.g., Abbasi et al. (2016) to investigate actual scenarios as to how the potential of data and analytics might be shaped to form entrepreneurial competitive advantages.

Furthermore, we contribute to a deeper conceptual understanding of how analytics might be leveraged in service offerings depending on the intended use. Previous research tends to demonstrate distinct approaches like machine learning from a technical perspective, but rarely derives generalizable and strategic insights regarding its application in services (Hinz et al., 2019). Thus, our findings contribute to literature at the intersection of information systems and (strategic) service management by providing a better understanding of how analytics applications afford novel service offerings (Günther et al., 2017; Ostrom et al., 2015).

The proposed systematization provides insight into how and why respective ABS are designed to expand the service portfolio in real-world situations. Our theorizing attempt on ABS archetypes represents a type II mid-range theory (“theory for explaining”) (Gregor, 2006). While we do not claim to generate testable propositions concerning how ABS should look like, our analysis, instead, provides a theoretical conceptualization, which is “new and interesting […] to explain something that was poorly or imperfectly understood beforehand” (Gregor, 2006, p. 625). Thus, we believe the findings provide a promising reference point for further studies aiming to theorize how analytics applied to data might be of real use for customer-facing business practices.

This study also offers managerial implications that can be particularly helpful for organizations that are already taking advantage of ABS to create new customer value or are planning to do so. First, our quantitative analysis reveals typical approaches for using ABS to expand the service portfolio. This overview may help to establish a more informed and systematic development of strategies to use data and analytics in service offerings, in general. Second, each identified type of ABS is described to reveal commonalities and key components. This might provide a valuable orientation for organizations when investing in ABS initiatives, e.g., as possible blueprints for developing new services or guidelines for transforming existing ABS into more sophisticated ones. Third, this study reveals two essential drivers shaping ABS configuration and strategic evolvement in practice. We believe that carefully incorporating these key factors may inform decision-making during the development or evolvement of ABS in practice—ultimately leading to more sophisticated service design practices that seek to expand service portfolios with ABS.

### Limitations and future research

Our research certainly comes with some *limitations*. First, our analysis solely builds on start-up use cases. While we argue that start-ups are a purposeful source to identify ABS offerings, this decision limits the generalizability of our results regarding larger organizations. Second, our data collection approach is limited to the AngelList database and, therefore, this choice might influence the results’ generalizability as well. We were only able to consider ABS by start-ups that tout for investors on this platform, increasing the chance to miss innovative ABS elsewhere. Third, the data sample size in this study was limited to 105 use cases due to the significant amount of manual work required to code each case. While this reflects the exploratory nature of our research, this decision potentially limits the ability to identify more nuanced differences between use cases using cluster analysis. Finally, our archetype triangulation is based on interviews with decision-makers from seven different start-ups offering ABS. According to Eisenhardt (1989), this represents a sufficient number of cases to generate first theoretical insights with empirical grounding. However, this leaves potential for further analysis to dive deeper into each ABS archetype with a larger sample size to identify more nuanced characteristics.

These limitations at the same time leave the potential for *future research*. First, the analyses should be conducted again using a larger sample of cases, ideally including ABS use cases from larger organizations to increase the data sample’s diversity. Second, future research could more deeply investigate the causal effects of ABS-enriched service portfolios. For instance, it would be interesting to investigate the organization’s different business capabilities required depending on the archetype they intend to offer. As we already pointed out, the skillset regarding analytics capabilities seems to vary between the archetypes, making it a fruitful topic to start with. Third, we see the monetization of ABS as a promising field for future research. Interestingly, we found that organizations that had evolved, e.g., from archetype A to archetype B, usually kept their initial service offering and used it as a “basic service”, often in combination with a freemium revenue model to attract possible customers. Thus, future research might investigate how revenue models look like for different ABS archetypes. Lastly, we noticed a strong customer relationship as a prerequisite to offer sophisticated and individualized ABS (cf. archetypes C and D). Thus, we believe it would be interesting to further investigate how ABS providers may purposefully design desirable interactions and touchpoints with their customers. Related work of customer intimacy in the context of services and information systems (e.g., Habryn et al., 2012) might constitute a promising starting point to ultimately strive for a deeper theorizing process regarding the understanding of interactions and value co-creation mechanisms in ABS.
